# Air Trapping on Chest CT Is Associated with Worse Ventilation Distribution in Infants with Cystic Fibrosis Diagnosed following Newborn Screening

**DOI:** 10.1371/journal.pone.0023932

**Published:** 2011-08-19

**Authors:** Graham L. Hall, Karla M. Logie, Faith Parsons, Sven M. Schulzke, Gary Nolan, Conor Murray, Sarath Ranganathan, Phil Robinson, Peter D. Sly, Stephen M. Stick, on behalf of AREST CF

**Affiliations:** 1 Respiratory Medicine, Princess Margaret Hospital for Children, Perth, Australia; 2 School of Paediatrics and Child Health, University of Western Australia, Perth, Australia; 3 Telethon Institute for Child Health Research and Centre for Child Health Research, University of Western Australia, Perth, Australia; 4 School of Women's and Infant's Health, University of Western Australia, Perth, Australia; 5 Diagnostic Imaging, Princess Margaret Hospital for Children, Perth, Australia; 6 Respiratory Medicine, Royal Children's Hospital, Melbourne, Australia; 7 Murdoch Children's Research Institute, Melbourne, Australia; 8 Department of Paediatrics, University of Melbourne, Australia; 9 Queensland Children's Medical Research Institute, University of Queensland, Brisbane, Australia; 10 University Children's Hospital Basel, Basel, Switzerland; Hospital for Sick Children, Canada

## Abstract

**Background:**

In school-aged children with cystic fibrosis (CF) structural lung damage assessed using chest CT is associated with abnormal ventilation distribution. The primary objective of this analysis was to determine the relationships between ventilation distribution outcomes and the presence and extent of structural damage as assessed by chest CT in infants and young children with CF.

**Methods:**

Data of infants and young children with CF diagnosed following newborn screening consecutively reviewed between August 2005 and December 2009 were analysed. Ventilation distribution (lung clearance index and the first and second moment ratios [LCI, M_1_/M_0_ and M_2_/M_0_, respectively]), chest CT and airway pathology from bronchoalveolar lavage were determined at diagnosis and then annually. The chest CT scans were evaluated for the presence or absence of bronchiectasis and air trapping.

**Results:**

Matched lung function, chest CT and pathology outcomes were available in 49 infants (31 male) with bronchiectasis and air trapping present in 13 (27%) and 24 (49%) infants, respectively. The presence of bronchiectasis or air trapping was associated with increased M_2_/M_0_ but not LCI or M_1_/M_0_. There was a weak, but statistically significant association between the extent of air trapping and all ventilation distribution outcomes.

**Conclusion:**

These findings suggest that in early CF lung disease there are weak associations between ventilation distribution and lung damage from chest CT. These finding are in contrast to those reported in older children. These findings suggest that assessments of LCI could not be used to replace a chest CT scan for the assessment of structural lung disease in the first two years of life. Further research in which both MBW and chest CT outcomes are obtained is required to assess the role of ventilation distribution in tracking the progression of lung damage in infants with CF.

## Introduction

The non-invasive assessment of respiratory function in infants and young children with cystic fibrosis (CF) remains a significant challenge. The introduction of computed tomography (CT) scans of the chest has improved our understanding of lung damage in early CF lung disease [1], [2]. Reports of chest CT scans in infants and preschool children with CF are limited; however lung damage has been reported to be associated with infection and inflammation [3] and decreased lung function [4]. In infants and young children diagnosed with CF following newborn screening (NBS) we reported the presence of bronchiectasis soon after diagnosis with the presence and extent of lung damage being associated with increasing neutrophilic inflammation and the presence of *Pseudomonas aeruginosa* [5]. However, chest CT scans in infants and young children require a general anaesthetic and consideration of the subsequent life time radiation dose may preclude its regular use [6]. Therefore, surrogate techniques that reflect lung damage and that compliment objective measures by chest CT and that can be used for clinical management and as endpoints in clinical trials will be important developments for CF care.

The multiple breath washout technique (MBW) is an easily applied method that allows the assessment of functional residual capacity (FRC) and ventilation distribution indices such as the lung clearance index (LCI) and moment ratios. Assessments of LCI have been reported in neonates [7], [8], [9], infants [10], [11] and preschool children [12], [13], [14]. Studies of LCI in CF lung disease suggest the majority of preschool and school aged children have abnormal LCI [12], [13], [15], [16], [17], [18] while reports in infants with CF suggest lower prevalence of abnormal LCI [10]. The LCI has been reported to be increased with *P aeruginosa* infection in young children [12] and to decrease following intravenous antibiotic treatment for pulmonary exacerbation [19] and following treatment with inhaled hypertonic saline [20] and dornase alfa [21] in school aged children. To date there are scarce data reporting the relationship between LCI and structural lung damage in children with CF, with the authors unaware of published studies in infants with CF. In a retrospective analysis Gustafsson *et al*. [16] reported significantly increased LCI with the presence and extent of structural lung disease assessed from chest CT in school aged children and these findings have been replicated in two subsequent prospective studies [15], [18]. In these studies LCI was more sensitive than spirometry to detect the presence of structural lung damage with the authors concluding that an abnormal LCI was associated with an abnormal chest CT score. The aim of this study was to assess the relationships between structural lung damage as assessed by chest CT and ventilation distribution in infants and young children with CF diagnosed following newborn screening.

## Methods

### Subjects

Infants enrolled in the AREST CF early surveillance program and attending their annual review at Princess Margaret Hospital in Perth, Australia were eligible to be included in the analysis. Full details of the AREST CF early surveillance program have been published previously [5], [22]. Briefly, following identification through NBS and confirmation by genetic and/or sweat testing infants attend the CF clinic as soon as practical after diagnosis, the median age of attendance following diagnosis is 3.6 months [22]. The program includes infant lung function testing 2–3 days prior to a chest CT and bronchoalveolar lavage (BAL) as described below. Follow-up assessments are then undertaken annually. Chest CT was introduced into the AREST CF program in August 2005. The cross-sectional data presented here are from infants that attended for an annual review and were diagnosed with CF following NBS. Each infant was included in the analysis once using their first visit between August 2005 and 31 December 2009 that had complete results for ventilation distribution outcomes from MBW, chest CT outcomes and microbiology results from the BAL. The AREST CF program has ethical approval from the Princess Margaret Hospital for Children ethics committee and all parents/legal guardians signed written consent prior to enrollment.

### Infant lung function testing

Infant lung function testing was performed following oral chloral hydrate (60–100 mg/kg) sedation. Multiple breath washout testing was performed as previously described by our group [11] and others [7], [8], [9], [23], [24] using 5% sulphur-hexafluoride (SF6) as a tracer gas using an ultrasonic flow meter (Ecomedics AG, Duerten, Switzerland). Assessments of lung volume using this technique have been reported to show acceptable agreement when compared to mass spectrometry [25]. Data were included for analysis if there was no evidence of leak or irregular breathing, corrected using an updated temperature model [7], [23], [24] and the effective dead space of the measurement apparatus and face mask [26]. Functional residual capacity (FRC), LCI and the first and second moment ratios (M_1_/M_0_ and M_2_/M_0_) were derived as reported previously [8], [27].

### Chest CT and BAL

The chest CT followed by BAL were performed under the same intravenous general anesthesia protocol as previously described [5], [22]. Limited dose three slice chest CT scans (100 kV and 40–80 mAs) were obtained at a positive pressure of 25 cmH_2_0 and end-expiration (0 cmH_2_0). In March 2007 volumetric helical inspiratory scans (120 kv; 25 mAs; pitch 0.6; total radiation dose = 0.74–0.80 mSv) were introduced as the standard imaging technique, expiratory scans remained unchanged. In those children with a helical scans, three slices equivalent to the chest CT limited scan were extracted using the scout film to identify the “slices” to exactly correspond to the anatomical landmarks used for the limited slice scans. This strategy was used to maximise the number of scans available to compare with MBW outcomes. Immediately following chest CT the bronchoscope was introduced into the lower airway and BAL obtained from three aliquots of 1 mL/kg of sterile saline instilled into the right middle lobe followed by a single instillation into lingual or worst lobe on radiology. The two first aliquots (one right, one lingual) were sent separately for microbiological assessment and the 2^nd^ and 3^rd^ right middle lobe aliquots pooled and processed within 1 hour for subsequent inflammatory analysis. BAL samples were cultured by standard techniques and infection with a specific organism was considered as ≥10^5^ cfu/mL. Samples that cultured mixed oral flora (MOF) and isolated colonies <10^5^ CFU/mL were classified as uninfected.

### Chest CT scoring

Chest CT images were scored by a single experienced pediatric thoracic radiologist (C.M.) in six zones (upper, mid, and lower; right and left) as previously reported by our group [5], [22]. The presence of bronchiectasis or air-trapping was recorded in a binary fashion for each abnormality for each zone. If present, abnormalities were weighted on the proportion of affected airways in each zone for bronchiectasis (<50% = 1, >50% = 2) and area of each zone affected for air-trapping (<50% = 1, >50% = 2), as an indication of the extent of each abnormality. Thus the maximum score of 12 would represent over 50% of all lobes were affected. Bronchial wall thickening was not assessed in this analysis as we have previously shown that the intra-observer repeatability of bronchial wall thickening was low (62%; kappa = 0.237) compared to bronchiectasis (83%; kappa = 0.64) and air trapping (78%; kappa = 0.55) [5]. For the purposes of this analysis structural damage is reported as the presence (yes/no) and extent of each abnormality type.

### Statistical analysis

The primary objective of this analysis was to determine the relationships between outcomes from the MBW and the presence and extent of structural damage as assessed by chest CT. Differences in ventilation distribution outcomes and the presence (yes/no) of structural lung disease were assessed using non-parametric Mann-Whitney comparisons. Relationships between MBW outcomes and the chest CT extent scores were explored with Spearman correlations. We have previously reported that lung damage on chest CT was associated the presence of *Pseudomonas aeruginosa* [5] while recent conference reports have suggested an age related decline in LCI. Therefore we performed a multivariate regression analysis that included MBW outcome as the dependant variable and age (in weeks), infection status (uninfected and infected as described above) and extent of chest CT damage as independent variables. Infection status and extent of chest CT damage were classed as ordinal variables, but treated as continuous variables in the regression analysis. The residuals of the regression analyses were plotted and inspected to confirm normal distribution. All analyses were performed in SPSS Version 19 and significance accepted at the level of p<0.050.

## Results

**Figure 1:**
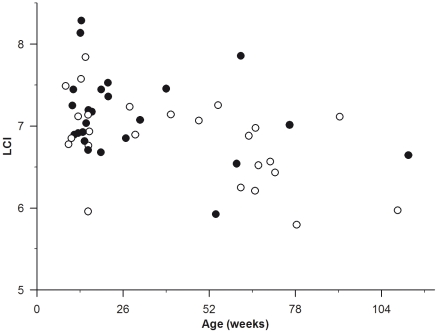
Lung clearance index (LCI) plotted against age (weeks) in infants diagnosed with cystic fibrosis following newborn screening. Infants with air trapping are shown in closed circles.

Forty-nine infants and young children (31 male) were included in the analysis with 26 (53%) children being homozygote for ΔF508. Six (12%) children had a bacterial infection at ≥10^5^ cfu/mL with 43 (88%) being classed as uninfected with 5 (10%) and 17 (35%) having isolated colonies or mixed oral flora, respectively. Bronchiectasis and air trapping was detected in 13 (27%) and 24 (49%) infants respectively. Twenty-two (45%) children had normal chest CT scans with no structural abnormalities, 10 (20%) children had both bronchiectasis and air trapping present, while 14 (29%) children had air trapping without bronchiectasis, with the remaining three (6%) children having bronchiectasis without air trapping. Full demographic, lung volume, ventilation distribution and chest CT outcomes are shown in table 1 with LCI against age shown in Figure 1.

**Table 1:** Anthropometric and clinical characteristics at the time of assessment.

Info	Mean±SD	Range
Age (weeks)	36.4±28.5	8.7–112.1
Body length (cm)	67.7±10.1	55–91
Body weight (kg)	7.73±2.84	3.69–14.0
Microbiology		
Uninfected	43 (88%)	
- Mixed Oral flora	17 (35%)	
- Isolate colonies	5 (10%)	
Infected	6 (12%)	
FRC (mL)	163.5±71.8	59.3–351.0
LCI	7.0±0.5	5.8–8.3
M_1_/M_0_	1.4±0.1	1.3–1.7
M_2_/M_0_	4.9±0.7	3.8–7.2
Presence of Bronchiectasis	13 (27%)	
Presence of Air Trapping	24 (49%)	
Extent of Bronchiectasis	0	0–4
Extent of Air Trapping	0	0–6

Continuous data expressed as mean ± SD (range). Categorical data expressed as absolute number (% proportion of cohort). Chest CT extent scores expressed as median (range). FRC: function residual capacity; LCI: lung clearance index: M_1_/M_0_: first to zeroth moment ratio; M_2_/M_0_: second to zeroth moment ratio.

**Figure 2:**
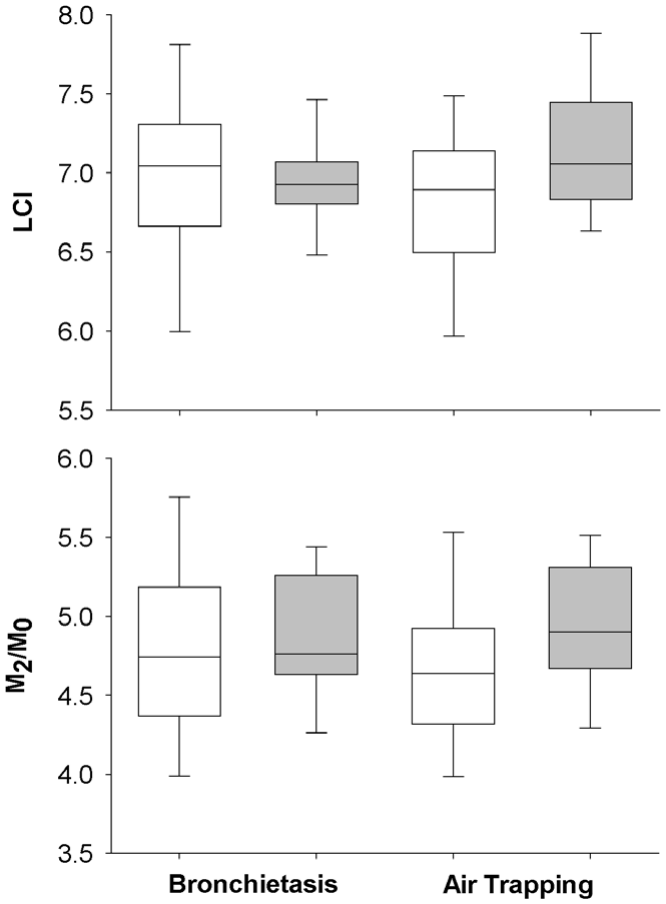
Lung clearance index (LCI; upper panel) and the second moment ratio (M_2_/M_0_; lower panel) difference with the presence and absence of bronchiectasis (n = 13 with and n = 36 without bronchiectasis) and air trapping (n = 24 with and n = 25 without air trapping) in infants with cystic fibrosis. Data are presented as box and whisker plots representing the 25^th^ and 75^th^ centiles and 10^th^ and 90^th^ centiles respectively. There were no differences in LCI between those infant with (grey box) and without (white box) lung damage while M_2_/M_0_ was significantly increased (p = 0.049) in those infants and young children with air trapping present on chest CT.

Lung clearance index was not significantly increased with the presence of bronchiectasis (7.0 (6.0–7.8) and 6.9 (6.4–7.5) bronchiectasis absent or present, respectively; p = 0.59 median (10–90^th^ centiles)) or air trapping (6.9 (6.0–7.5) and 7.1 (6.6–8.0) air trapping absent or present, respectively; p = 0.09). Similarly M_1_/M_0_ was not altered with the presence of structural lung damage (data not shown). In contrast M_2_/M_0_ was increased with the presence of air trapping (4.9 (4.2–5.6)) with compared to those without air trapping (4.6 (3.9–5.7); p = 0.049) but remain unchanged with bronchiectasis (4.7 (4.0–5.8) and 4.8 (4.1–5.5); bronchiectasis absent or present, respectively; p = 0.60). Changes in LCI and M_2_/M_0_ with the presence and absence of structural lung disease are shown in figure 2.

**Figure 3:**
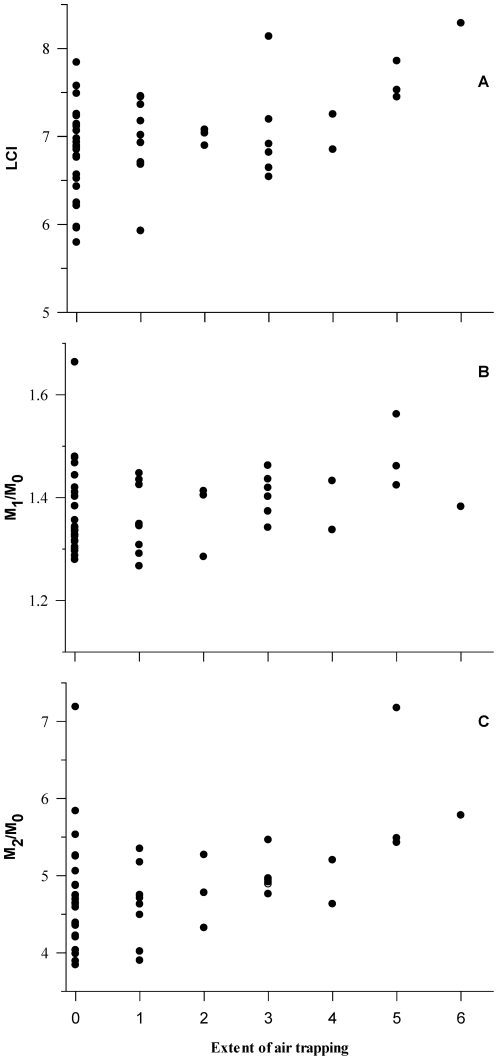
Changes in the lung clearance index (LCI; panel A) and the first (M_1_/M_0_; panel B) and second moment ratios (M_2_/M_0_; panel C) with the increasing extent of air trapping. Increasing extent of air trapping was associated with an increased LCI (r = 0.31 p = 0.03) and M_2_/M_0_ (r = 0.40; p<0.005) but not M_1_/M_0_ (r = 0.28; p = 0.051).

Univariate non-parametric correlations showed that there were no associations between ventilation distribution outcomes and the extent of bronchiectasis (data not shown). In contrast LCI (r = 0.31 p = 0.03) and M_2_/M_0_ (r = 0.40; p<0.005) but not M_1_/M_0_ (r = 0.28; p = 0.051) were significantly increased with increasing extent of air trapping (Figure 3).

After controlling for age and infection status in the multivariate regression analysis there were no associations between ventilation distribution outcomes and the extent of bronchiectasis (data not shown). In contrast LCI, M_1_/M_0_ and M_2_/M_0_ significantly increased with increasing extent of air trapping (Table 2).

**Table 2:** Multivariate relationships between ventilation inhomogeneity and the extent of air trapping.

	LCI	M_1_/M_0_	M_2_/M_0_
Age (weeks)	**−0.01 [−0.01 −0.00]** *	0.00 [0.00 0.01]	0.00 [−0.01 0.01]
Infection	−0.15 [−0.57 0.27]	0.04 [−0.07 0.08]	0.13 [−0.48 0.73]
Air trapping	**0.12 [0.04 0.21]** *	**0.01 [0.00 0.03]** #	**0.16 [0.04 0.28]** ∑

LCI: lung clearance index; M_1_/M_0_: first to zeroth moment ratio; M_2_/M_0_: second to zeroth moment ratio. Infection was categorised as ≥10^5^ colony forming units. The extent of lung damage was assessed on a scale of 0 (no damage) to 12 (>50% damage in all lobes). Regression coefficients and 95% confidence intervals are shown.

∑ p<0.01;

*p<0.005;

# p<0.05.

## Discussion

We report the relationships between ventilation distribution derived from the multiple breath washout technique and the presence and extent of structural lung disease in infants and young children diagnosed with CF following newborn screening. In this study the presence and extent of bronchiectasis was not associated with ventilation distribution assessed by LCI. In contrast the presence of air trapping on the chest CT was associated with an increase in M_2_/M_0_, but not LCI or M_1_/M_0_. After controlling for age and infection status the extent of air trapping was associated with increases in all reported ventilation distributed outcomes. Our results suggest that in early CF lung disease assessments of ventilation distribution are only weakly associated with lung damage on chest CT and may not have the same role as in older children and adults. This study does not support the use of LCI to replace a chest CT scan for the assessment of structural lung disease in the first two years of life.

Previous studies in school-aged children with CF have reported abnormal LCI to be associated with the presence and extent of structural lung damage [15], [16], [18]. In a retrospective cross-sectional analysis Gustafsson *et al*. [16] reported abnormal LCI in 25 of 27 children with abnormal HRCT scores while normal LCI and a normal HRCT were reported in 11 of 17 children giving a concordance of 82%. Similarly, prospective cross-sectional studies from Ellemunter *et al*. and Owens *et al*. reported concordances of 81–82% [15], [18]. Considered together these studies indicate that lung damage in school aged children is associated with a significantly increased LCI and in clear contrast with the results reported in this study. The differences between this study and these reports may be due to a number of reasons. The children in these studies were of school age and cooperative, in contrast to the current study in infants whom are unable to cooperate with lung function testing. Older children complete MBW testing sitting and awake, while in infants lung function testing is performed in the supine position following sedation. Similarly chest CT in infants and young children is performed under general anaesthesia while older children are awake and cooperative during the CT scan. Further in this study infant lung function was conducted two to three days prior to the chest CT and in contrast to studies in older cooperative children in whom all assessments can be made during the same visit. We cannot discount that these methodological differences may impact on the ability of markers of ventilation distribution such as LCI to reflect structural lung damage in infants with CF. However, as the methods used here (i.e. supine and sedated infant lung function and chest CT under general anaesthesia) are standard methods for use in infants we do not believe that these methodological differences alter the conclusions of this study nor on the generalisation of these results to other studies in infants with CF. The current study is in infants and young children in whom structural lung disease is significantly less than that of school-aged children. The studies described above in older children (6 years of age and older) reported between 61 and 85% of children to have abnormal chest CT with 25 to 43% of children having current or chronic *Pseudomonas aeruginosa* [15], [16], [18]. In contrast in this study 55% (n = 25) children had some form of structural lung damage of which 10 (20%) had both bronchiectasis and air trapping. Similarly infection with airway pathogens was low with only 6 (12%) infants classed as infected of which none were colonised with *Pseudomonas aeruginosa*. Considered together these factors confirm that lung disease in infants with CF is less advanced than that of school aged children and reinforce the importance of not extrapolating research findings from older age groups to infants and young children.

Studies in older children have reported abnormal LCI values in 70 to 95% of children [15], [16], [18], [28]. In contrast to older children and adults there are relatively few studies reporting LCI in infants with CF. In infants with CF diagnosed following clinical presentation Lum *et al* [10] reported 56% with abnormal LCI (LCI>7.8) when compared to healthy infants. Preliminary data from the same group indicate a lower prevalence of abnormal LCI of 25% (5 of 20) in infants diagnosed with CF following NBS suggesting that lower disease severity in young infants compared to older infants [29]. Interestingly the upper limit of normal in the younger infants was ∼8.2 compared to 7.8 in older infants aged up to two years of age suggesting a decrease in LCI with age and in agreement with the decline in LCI with age in this study (figure 1). The only other study reporting ventilation distribution in infants diagnosed with CF following NBS is from Belessis [30]. This study reported abnormal LCI in 15 of 47 (32%) infants with CF when compared to local healthy controls studied in the same laboratory with an upper limit of normal of 7.41. Considered together these studies suggest that lung disease in infants with CF is milder than that seen in older children as assessed by LCI.

A limitation of the current study is that we do not have local healthy controls and therefore we are unable to make definitive statements regarding the prevalence of abnormal LCI in our study. The methods, equipment and tracer gas used by Belessis [30] are identical to those used in this study. Using an upper limit of normal of 7.41, 10 (20%) of the infants reported in this study have an abnormal LCI. Infants with an abnormal LCI had an increase in the extent of air trapping (2: 0–5.9: median: 10–90^th^ centiles) when compared to infants with a normal LCI (0: 0–3; Mann-Whitney U test: p = 0.03). In contrast there was no different in the extent of bronchiectasis in infants with abnormal LCI (0: 0–1) when compared to infants with a normal LCI (0:0–2: p = 0.43). Currently no other study in infants with CF has reported moment ratios and the upper limits of normal are unknown. Similarly, the clinically relevant difference in LCI and moment ratios in infants has not been reported and therefore we are unable to speculate on the clinical relevance of the associations between the lung damage and ventilation distribution reported here. There is an urgent need for multi-centre studies of MBW outcomes in infants from which robust reference equations for ventilation distribution outcomes can be derived.

While between centre differences preclude definitive statements on the relationship between abnormal LCI and the presence of lung damage on chest CT in infants with CF, we can clearly state that LCI and moment ratios are not increased with the presence of bronchiectasis in our population of infants and young children diagnosed with CF following NBS. There were significant but weak increases in ventilation distribution with increasing extent of air trapping in the present study. Given that the range of LCI and moment ratio values in those infants and young children with lung damage was within that of individuals without lung damage (Figure 3) it is evident that assessments of ventilation distribution in early life are unlikely to be useful as a screening tool to identify those infants with lung damage as has been suggested in studies in school aged children [15], [16], [18]. Another limiting factor of the present study was the use of a three-slice chest CT scan which may over or under-estimate the presence of bronchiectasis and structural lung disease [31]. In March 2007 the AREST CF collaboration commenced the use of low-dose volumetric chest CT scans. The limited data from volumetric scans confirmed the complete overlap of values of LCI for those children with and without structural lung disease (data not shown) and suggest that the results reported here are not significantly influenced by the chest CT method used.

Lum et al reported no associations between mode of diagnosis, clinical history of recent symptoms and LCI in infants diagnosed following clinical presentation [10]. In infants diagnosed with CF following NBS Belessis reported increased LCI in infants infected with *Pseudomonas aeruginosa* (n = 7; LCI = 7.66: 7.26–8.48: median: inter-quartile range) when compared to infants infected with other organisms (n = 40; LCI = 7.03: 6.56–7.41: p = 0.007). The primary aim of this analysis was to investigate the relationship between structural lung damage and ventilation distribution outcomes and as such this analysis was not powered to assess the impact of infection or other potentially relevant clinical factors such as mutation type, nutritional status or presence of symptoms on ventilation distribution outcomes. Further studies with larger number of infants with CF with a broader range of lung disease are required to investigate the impact of these clinical outcomes on ventilation distribution in infants and young children with CF.

The London CF collaboration recently demonstrated that LCI measured between three and five years of age tracks through to school age suggesting that measurements of LCI in early life may assist in identifying the early onset of CF lung disease [13]. The associations reported here may represent the initial stages of the link between abnormal LCI and lung damage seen in older children and further longitudinal studies following the comparative evolution of LCI and lung damage until school-age are needed so as to provide further information on the early development of an abnormal LCI and its potential to predict an abnormal chest CT in the early school years.

In summary we report that the lung clearance index and moment ratios derived from the multiple breath washout technique cannot identify the presence of structural lung disease in infants and young children diagnosed with cystic fibrosis following newborn screening. The current study does not support the use of LCI as a replacement or surrogate outcome for lung damage assessed by chest CT in early life. Further research in which both ventilation distribution and chest CT outcomes are obtained in larger groups of infants with CF are required to better define the role of ventilation distribution outcomes for monitoring disease progression and as an outcome for clinical trials.
